# Communities, policy-makers and scientists: a critical partnership for malaria elimination

**DOI:** 10.1186/1475-2875-11-S1-O14

**Published:** 2012-10-15

**Authors:** Halima A Mwenesi

**Affiliations:** 1 African Leaders Malaria Alliance Secretariat, Dare Salaam, Tanzania; 2 Global Health, Population and Nutrition Group, FHI360, 1825 Connecticut Avenue, Washington, DC, 20009, USA

## 

Partnership between all stakeholders involved in malaria efforts has been demonstrated to be a pillar of successful malaria control, a precursor for malaria elimination. Over the last decade, collaboration between donors, governments, academia, industry (private sector), civil society and to reasonable extent communities has borne fruits that were only a dream in the recent past.

Manuals about the prerequisites for countries moving from control to elimination abound. They point out the need for country programs to embrace a paradigm shift that entails not only thinking differently but also managing differently. Key to the move from control to elimination for a country is the sustained “free from local transmission” status for three years at least, thus having malaria off the list of a country’s key health problems, driven by a strong political commitment to follow through overall development efforts including the Millennium Development Goals, development of strong health systems and enactment of adequate policies for vigilance/surveillance together with enhanced legislation to support some of the policies. In addition, a cooperating epidemiological condition that allows positive parasitological rates of below established thresholds is critical.

This paper while acknowledging the importance of all partners in the malaria control equation, posits that the partnership between communities, policy-makers and scientists be strengthened. Communities that embrace and believe that malaria can be controlled are better able to be engaged in surveillance and vigilance, and are also better able to demand for services and follow through with utilization of necessary tools during the pre-elimination to elimination phase. This paper further argues that scientists who are the ones charged with providing evidence upon which policies and strategies are based have to ensure their evidence is unpacked to enable policymakers make rational decisions - based on science and not political rhetoric. Countries that have conditions that are conducive to moving to elimination should be supported and encouraged to do so. On the other hand, countries in which conditions for elimination are fraught with challenges including long or year-round transmission (high endemicity), low coverage of effective malaria control interventions and lack of adequate funding among others should be advised against taking the elimination route prematurely.

Examples of countries that have registered progress in Africa through deliberate strengthening of partnerships as described above will be provided. In addition, the paper will provide proposals about channels that can be used to reach policy-makers, who must of necessity not only steward their countries’ control to elimination agenda, but also provide strong leadership for inevitable cross-border collaboration.

